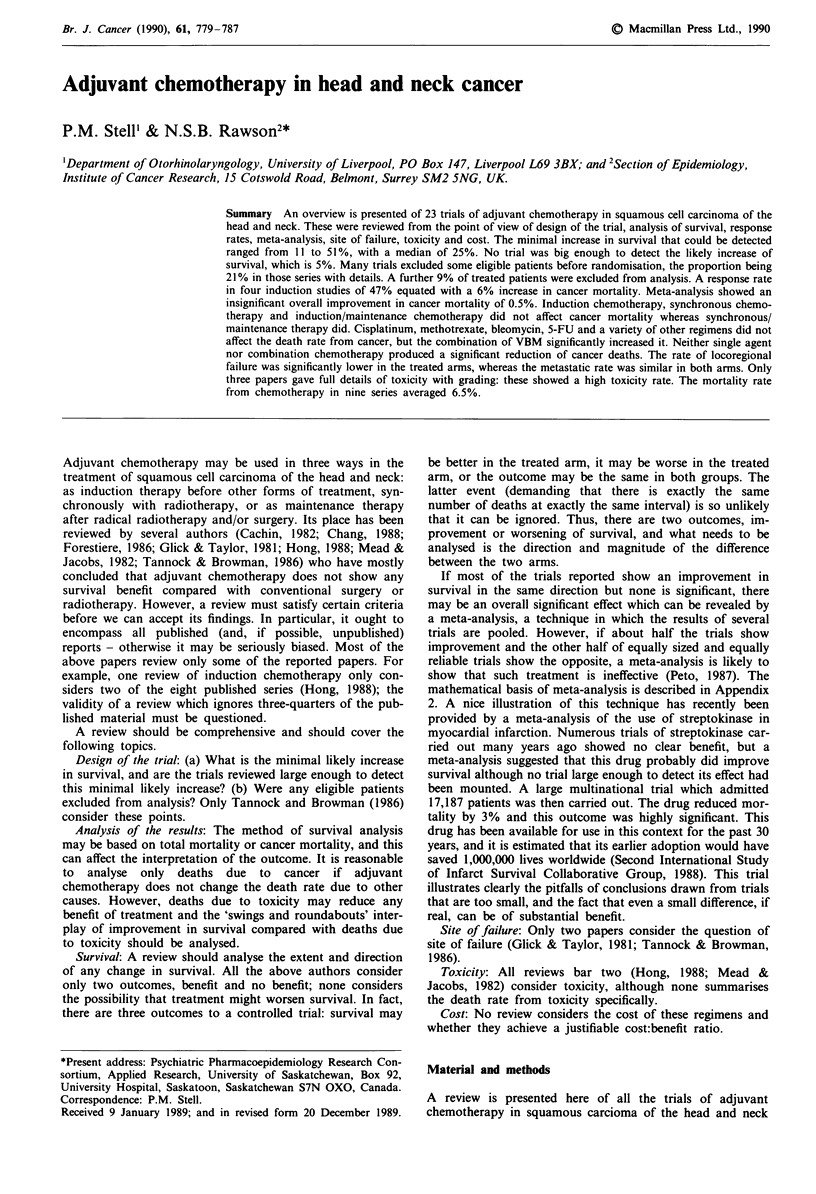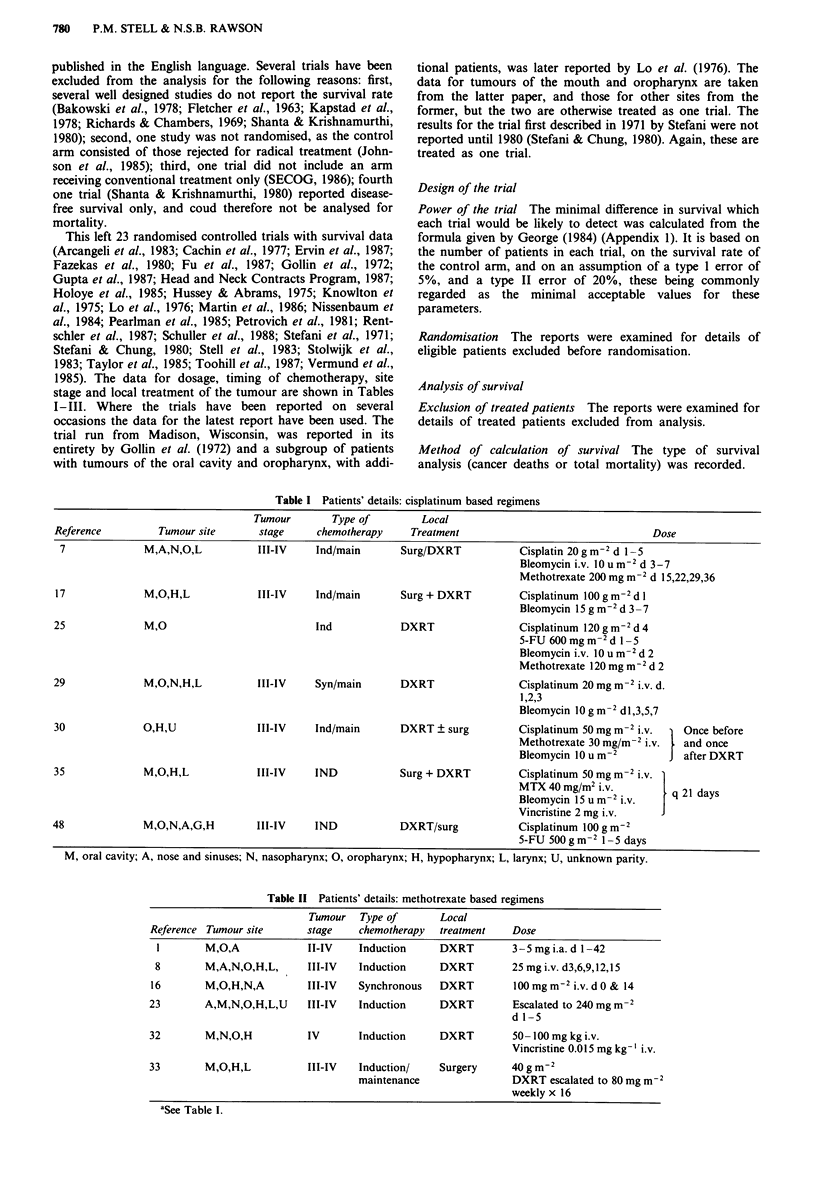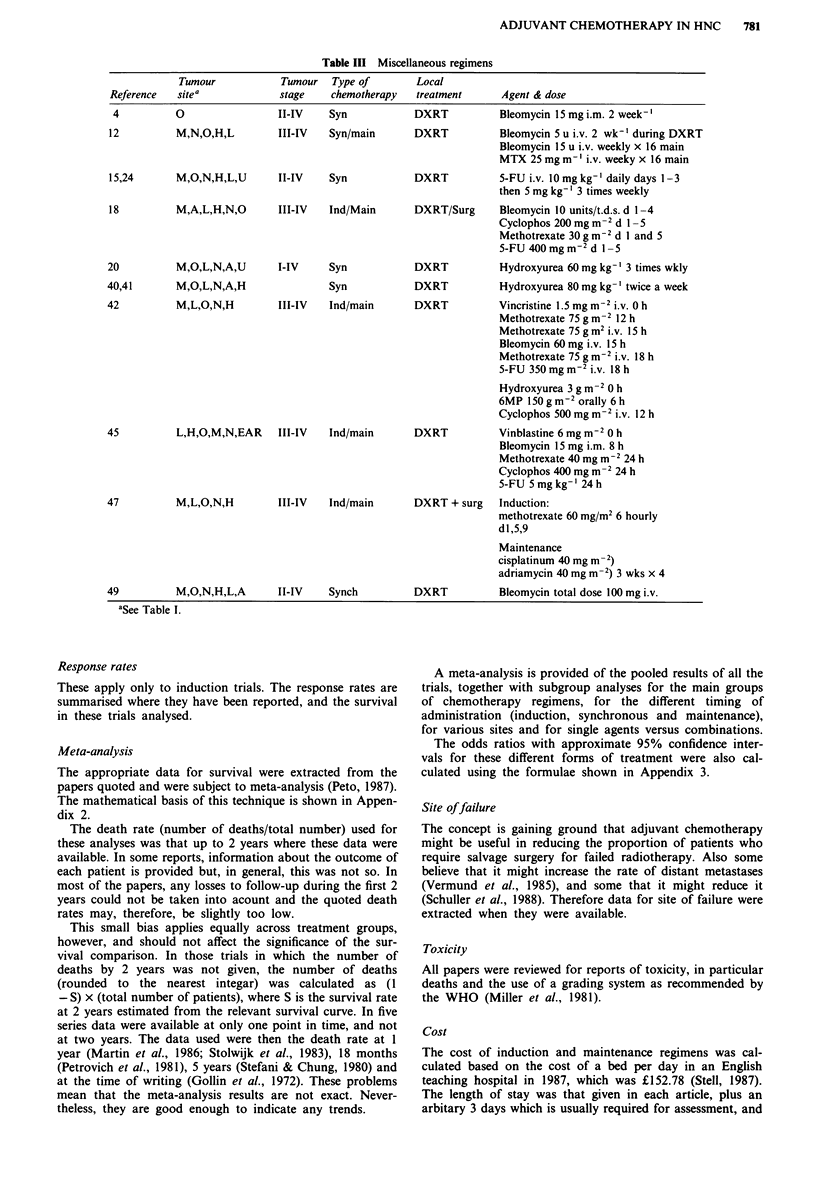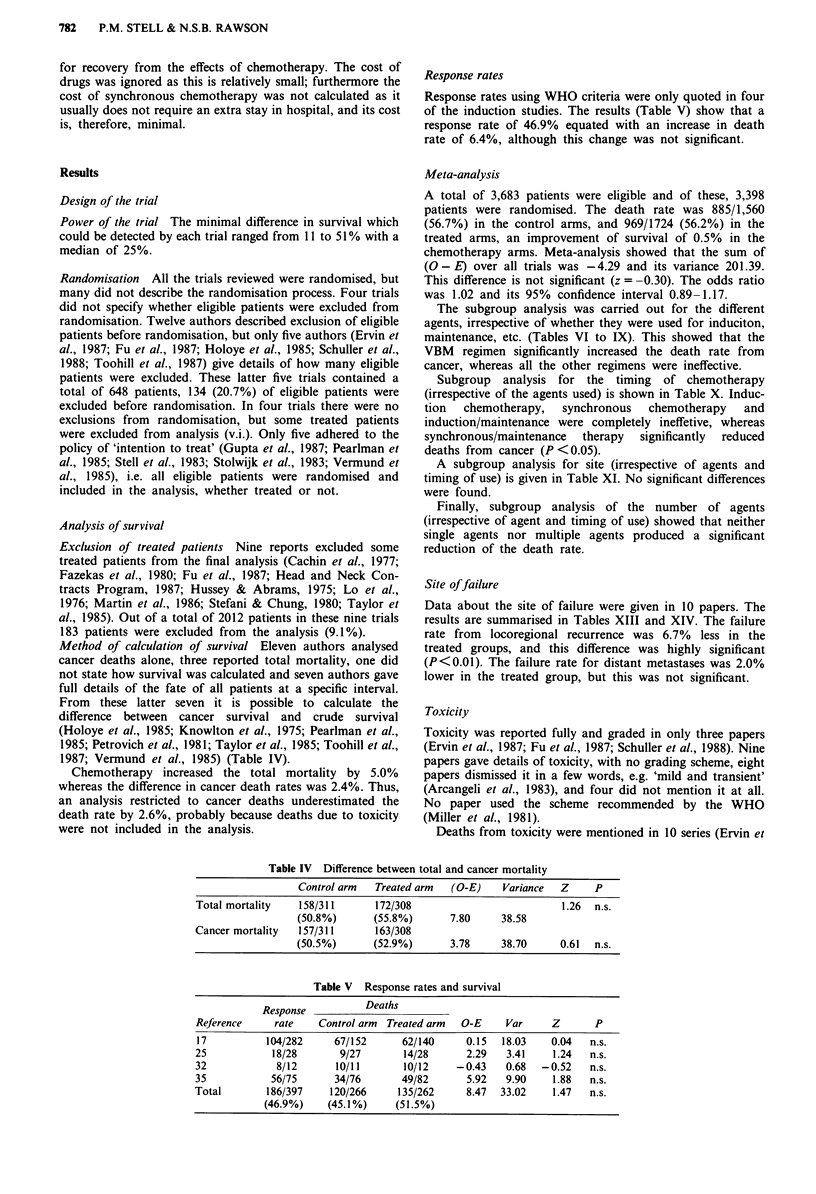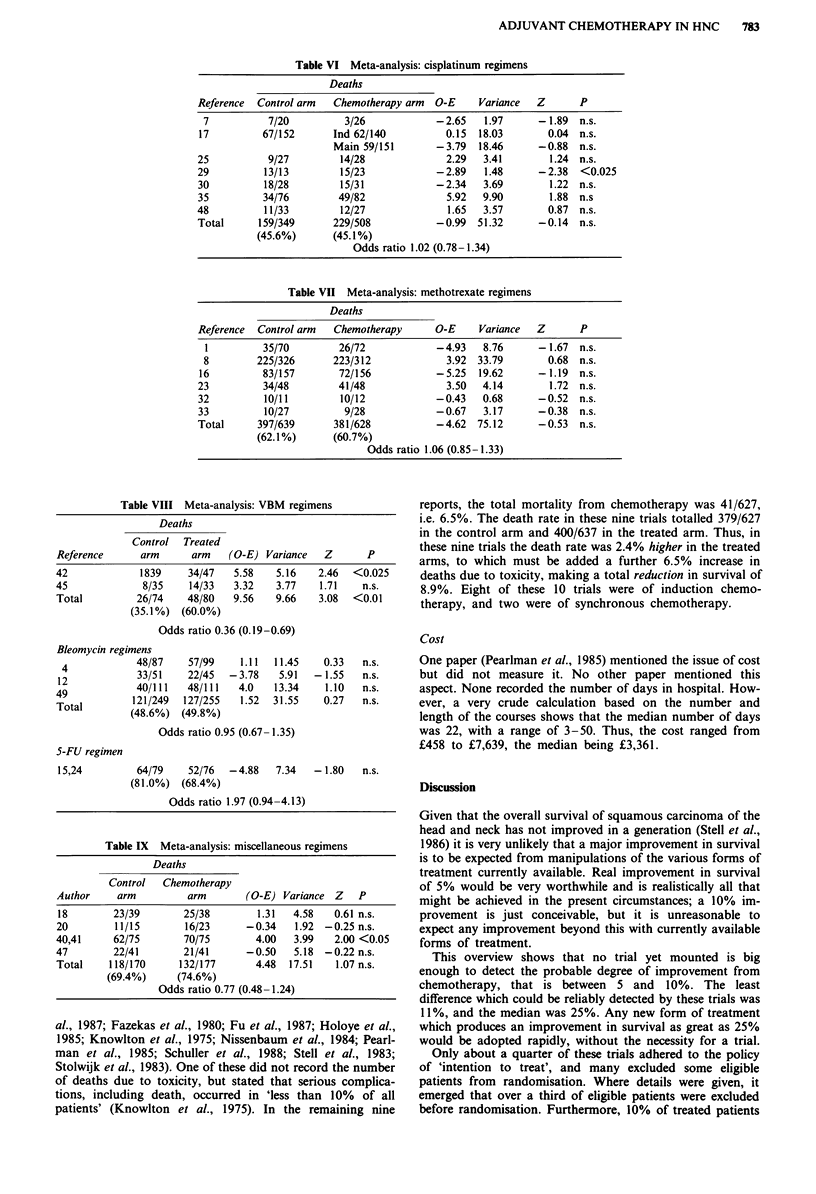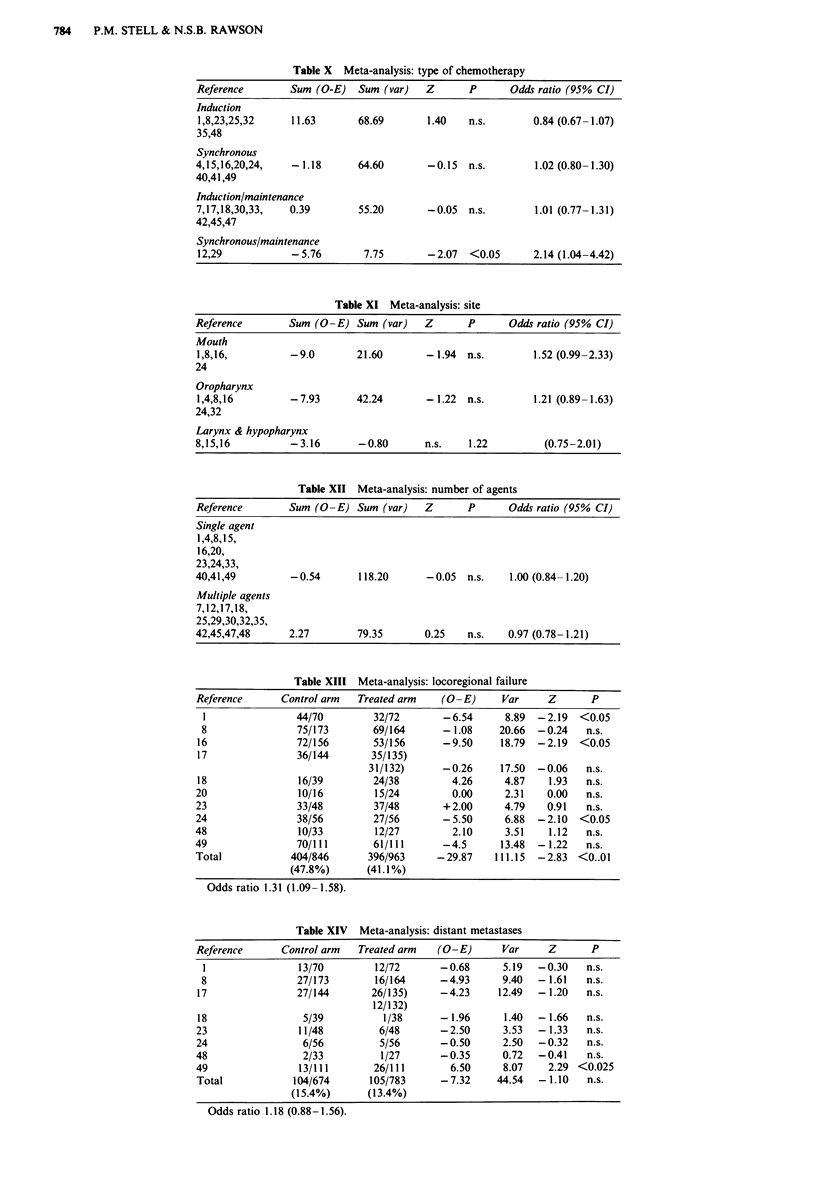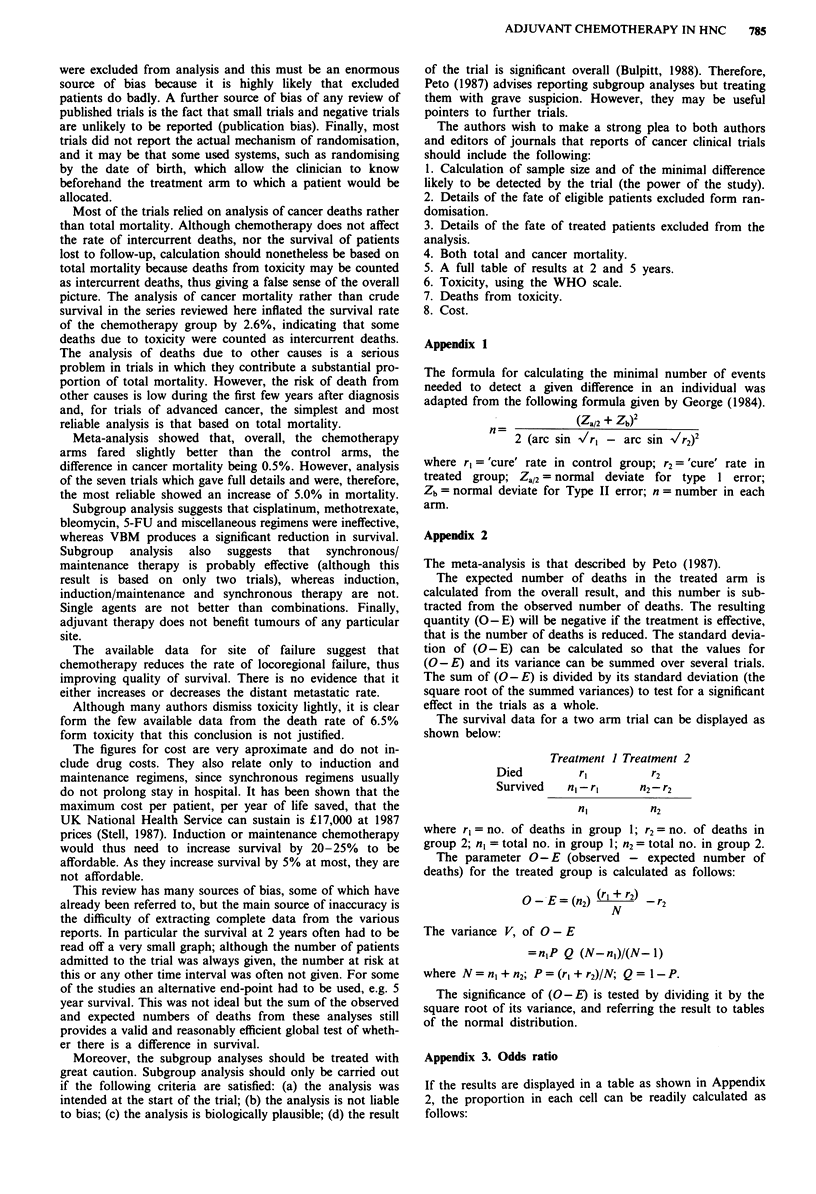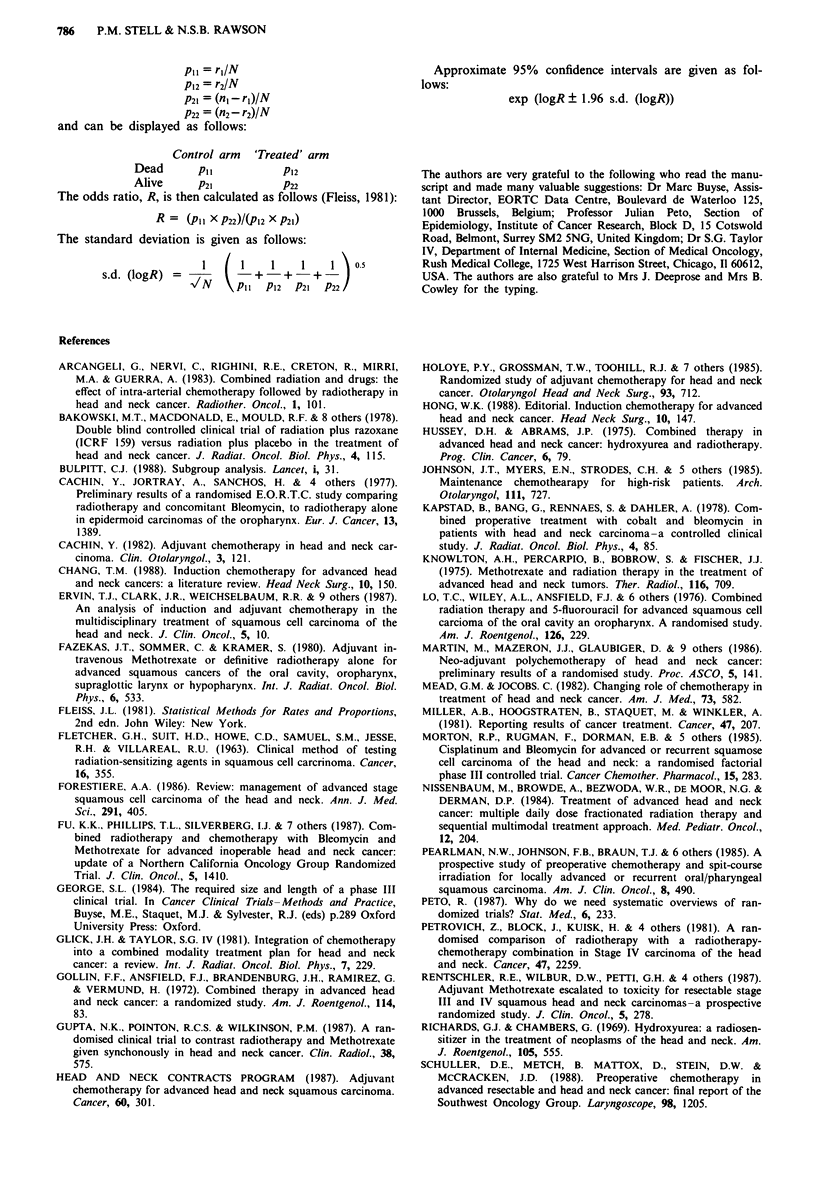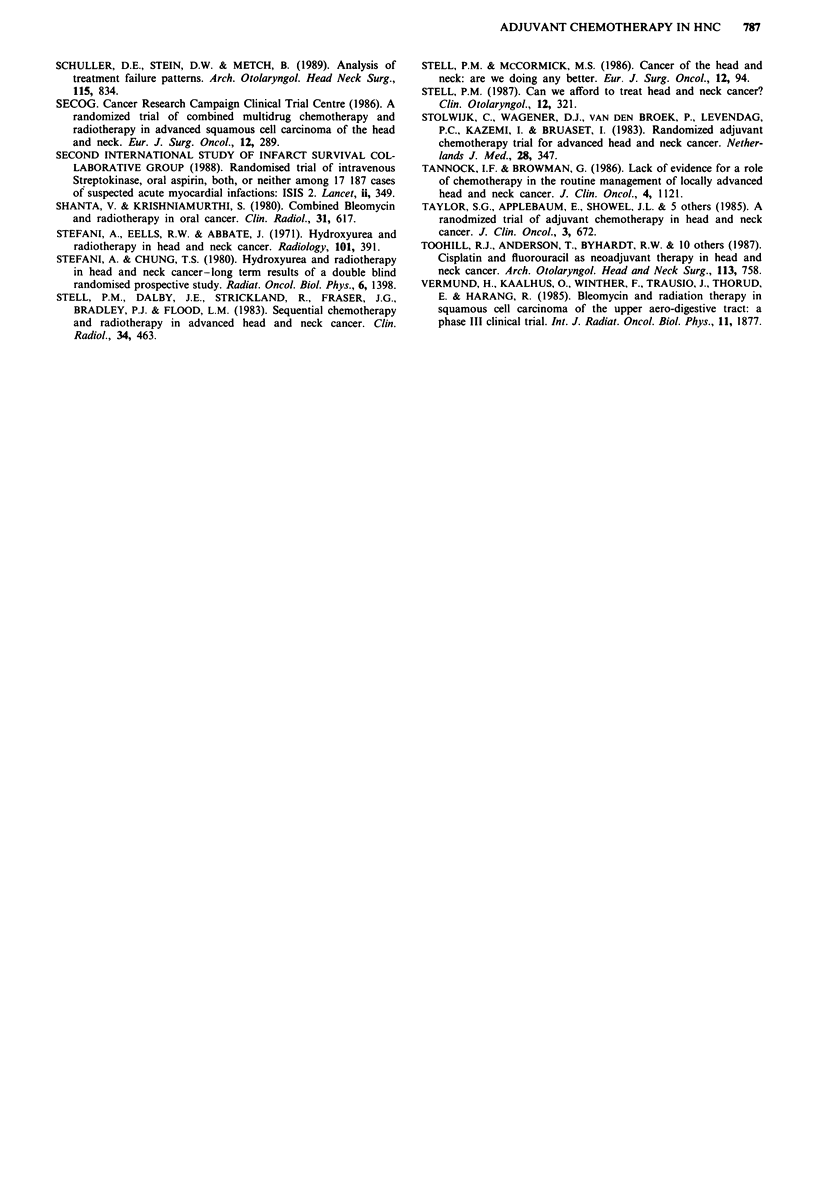# Adjuvant chemotherapy in head and neck cancer.

**DOI:** 10.1038/bjc.1990.175

**Published:** 1990-05

**Authors:** P. M. Stell, N. S. Rawson

**Affiliations:** Department of Otorhinolaryngology, University of Liverpool, UK.

## Abstract

An overview is presented of 23 trials of adjuvant chemotherapy in squamous cell carcinoma of the head and neck. These were reviewed from the point of view of design of the trial, analysis of survival, response rates, meta-analysis, site of failure, toxicity and cost. The minimal increase in survival that could be detected ranged from 11 to 51%, with a median of 25%. No trial was big enough to detect the likely increase of survival, which is 5%. Many trials excluded some eligible patients before randomisation, the proportion being 21% in those series with details. A further 9% of treated patients were excluded from analysis. A response rate in four induction studies of 47% equated with a 6% increase in cancer mortality. Meta-analysis showed an insignificant overall improvement in cancer mortality of 0.5%. Induction chemotherapy, synchronous chemotherapy and induction/maintenance chemotherapy did not affect cancer mortality whereas synchronous/maintenance therapy did. Cisplatinum, methotrexate, bleomycin, 5-FU and a variety of other regimens did not affect the death rate from cancer, but the combination of VBM significantly increased it. Neither single agent nor combination chemotherapy produced a significant reduction of cancer deaths. The rate of locoregional failure was significantly lower in the treated arms, whereas the metastatic rate was similar in both arms. Only three papers gave full details of toxicity with grading: these showed a high toxicity rate. The mortality rate from chemotherapy in nine series averaged 6.5%.


Br. J. Cancer (1990), 61, 779-787 © Macmillan Press Ltd., 1990

### Adjuvant chemotherapy in head and neck cancer

P.M. Stell[1] & N.S.B. Rawson[2]*

[1]*Department of Otorhinolaryngology, University of Liverpool, PO Box 147, Liverpool L69 3BX; and* [2]*Section of Epidemiology, Institute of Cancer Research, 15 Cotswold Road, Belmont, Surrey SM2 5NG, UK.*

**Summary** An overview is presented of 23 trials of adjuvant chemotherapy in squamous cell carcinoma of the head and neck. These were reviewed from the point of view of design of the trial, analysis of survival, response rates, meta-analysis, site of failure, toxicity and cost. The minimal increase in survival that could be detected ranged from 11 to 51%, with a median of 25%. No trial was big enough to detect the likely increase of survival, which is 5%. Many trials excluded some eligible patients before randomisation, the proportion being 21% in those series with details. A further 9% of treated patients were excluded from analysis. A response rate in four induction studies of 47% equated with a 6% increase in cancer mortality. Meta-analysis showed an insignificant overall improvement in cancer mortality of 0.5%. Induction chemotherapy, synchronous chemotherapy and induction/maintenance chemotherapy did not affect cancer mortality whereas synchronous/maintenance therapy did. Cisplatinum, methotrexate, bleomycin, 5-FU and a variety of other regimens did not affect the death rate from cancer, but the combination of VBM significantly increased it. Neither single agent nor combination chemotherapy produced a significant reduction of cancer deaths. The rate of locoregional failure was significantly lower in the treated arms, whereas the metastatic rate was similar in both arms. Only three papers gave full details of toxicity with grading: these showed a high toxicity rate. The mortality rate from chemotherapy in nine series averaged 6.5%.

Adjuvant chemotherapy may be used in three ways in the treatment of squamous cell carcinoma of the head and neck: as induction therapy before other forms of treatment, synchronously with radiotherapy, or as maintenance therapy after radical radiotherapy and/or surgery. Its place has been reviewed by several authors (Cachin, 1982; Chang, 1988; Forestiere, 1986; Glick & Taylor, 1981; Hong, 1988; Mead & Jacobs, 1982; Tannock & Browman, 1986) who have mostly concluded that adjuvant chemotherapy does not show any survival benefit compared with conventional surgery or radiotherapy. However, a review must satisfy certain criteria before we can accept its findings. In particular, it ought to encompass all published (and, if possible, unpublished) reports – otherwise it may be seriously biased. Most of the above papers review only some of the reported papers. For example, one review of induction chemotherapy only considers two of the eight published series (Hong, 1988); the validity of a review which ignores three-quarters of the published material must be questioned.

A review should be comprehensive and should cover the following topics.

*Design of the trial*: (a) What is the minimal likely increase in survival, and are the trials reviewed large enough to detect this minimal likely increase? (b) Were any eligible patients excluded from analysis? Only Tannock and Browman (1986) consider these points.

*Analysis of the results*: The method of survival analysis may be based on total mortality or cancer mortality, and this can affect the interpretation of the outcome. It is reasonable to analyse only deaths due to cancer if adjuvant chemotherapy does not change the death rate due to other causes. However, deaths due to toxicity may reduce any benefit of treatment and the 'swings and roundabouts' interplay of improvement in survival compared with deaths due to toxicity should be analysed.

*Survival*: A review should analyse the extent and direction of any change in survival. All the above authors consider only two outcomes, benefit and no benefit; none considers the possibility that treatment might worsen survival. In fact, there are three outcomes to a controlled trial: survival may be better in the treated arm, it may be worse in the treated arm, or the outcome may be the same in both groups. The latter event (demanding that there is exactly the same number of deaths at exactly the same interval) is so unlikely that it can be ignored. Thus, there are two outcomes, improvement or worsening of survival, and what needs to be analysed is the direction and magnitude of the difference between the two arms.

If most of the trials reported show an improvement in survival in the same direction but none is significant, there may be an overall significant effect which can be revealed by a meta-analysis, a technique in which the results of several trials are pooled. However, if about half the trials show improvement and the other half of equally sized and equally reliable trials show the opposite, a meta-analysis is likely to show that such treatment is ineffective (Peto, 1987). The mathematical basis of meta-analysis is described in Appendix 2. A nice illustration of this technique has recently been provided by a meta-analysis of the use of streptokinase in myocardial infarction. Numerous trials of streptokinase carried out many years ago showed no clear benefit, but a meta-analysis suggested that this drug probably did improve survival although no trial large enough to detect its effect had been mounted. A large multinational trial which admitted 17,187 patients was then carried out. The drug reduced mortality by 3% and this outcome was highly significant. This drug has been available for use in this context for the past 30 years, and it is estimated that its earlier adoption would have saved 1,000,000 lives worldwide (Second International Study of Infarct Survival Collaborative Group, 1988). This trial illustrates clearly the pitfalls of conclusions drawn from trials that are too small, and the fact that even a small difference, if real, can be of substantial benefit.

*Site of failure*: Only two papers consider the question of site of failure (Glick & Taylor, 1981; Tannock & Browman, 1986).

*Toxicity*: All reviews bar two (Hong, 1988; Mead & Jacobs, 1982) consider toxicity, although none summarises the death rate from toxicity specifically.

*Cost*: No review considers the cost of these regimens and whether they achieve a justifiable cost:benefit ratio.

### Material and methods

A review is presented here of all the trials of adjuvant chemotherapy in squamous carcioma of the head and neck

*Present address: Psychiatric Pharmacoepidemiology Research Consortium, Applied Research, University of Saskatchewan, Box 92, University Hospital, Saskatoon, Saskatchewan S7N OXO, Canada. Correspondence: P.M. Stell.
Received 9 January 1989; and in revised form 20 December 1989.

published in the English language. Several trials have been excluded from the analysis for the following reasons: first, several well designed studies do not report the survival rate (Bakowski *et al.*, 1978; Fletcher *et al.*, 1963; Kapstad *et al.*, 1978; Richards & Chambers, 1969; Shanta & Krishnamurthi, 1980); second, one study was not randomised, as the control arm consisted of those rejected for radical treatment (Johnson *et al.*, 1985); third, one trial did not include an arm receiving conventional treatment only (SECOG, 1986); fourth one trial (Shanta & Krishnamurthi, 1980) reported disease-free survival only, and coud therefore not be analysed for mortality.

This left 23 randomised controlled trials with survival data (Arcangeli *et al.*, 1983; Cachin *et al.*, 1977; Ervin *et al.*, 1987; Fazekas *et al.*, 1980; Fu *et al.*, 1987; Gollin *et al.*, 1972; Gupta *et al.*, 1987; Head and Neck Contracts Program, 1987; Holoye *et al.*, 1985; Hussey & Abrams, 1975; Knowlton *et al.*, 1975; Lo *et al.*, 1976; Martin *et al.*, 1986; Nissenbaum *et al.*, 1984; Pearlman *et al.*, 1985; Petrovich *et al.*, 1981; Rentschler *et al.*, 1987; Schuller *et al.*, 1988; Stefani *et al.*, 1971; Stefani & Chung, 1980; Stell *et al.*, 1983; Stolwijk *et al.*, 1983; Taylor *et al.*, 1985; Toohill *et al.*, 1987; Vermund *et al.*, 1985). The data for dosage, timing of chemotherapy, site stage and local treatment of the tumour are shown in Tables I–III. Where the trials have been reported on several occasions the data for the latest report have been used. The trial run from Madison, Wisconsin, was reported in its entirety by Gollin *et al.* (1972) and a subgroup of patients with tumours of the oral cavity and oropharynx, with additional patients, was later reported by Lo *et al.* (1976). The data for tumours of the mouth and oropharynx are taken from the latter paper, and those for other sites from the former, but the two are otherwise treated as one trial. The results for the trial first described in 1971 by Stefani were not reported until 1980 (Stefani & Chung, 1980). Again, these are treated as one trial.

### *Design of the trial*

*Power of the trial* The minimal difference in survival which each trial would be likely to detect was calculated from the formula given by George (1984) (Appendix 1). It is based on the number of patients in each trial, on the survival rate of the control arm, and on an assumption of a type 1 error of 5%, and a type II error of 20%, these being commonly regarded as the minimal acceptable values for these parameters.

*Randomisation* The reports were examined for details of eligible patients excluded before randomisation.

### *Analysis of survival*

*Exclusion of treated patients* The reports were examined for details of treated patients excluded from analysis.

*Method of calculation of survival* The type of survival analysis (cancer deaths or total mortality) was recorded.

**Table I** Patients' details: cisplatinum based regimens

| *Reference* | *Tumour site* | *Tumour stage* | *Type of chemotherapy* | *Local Treatment* | *Dose* | |
|---|---|---|---|---|---|---|
| 7 | M,A,N,O,L | III-IV | Ind/main | Surg/DXRT | Cisplatin 20 g m$^{-2}$ d 1–5<br>Bleomycin i.v. 10 u m$^{-2}$ d 3–7<br>Methotrexate 200 mg m$^{-2}$ d 15,22,29,36 | |
| 17 | M,O,H,L | III-IV | Ind/main | Surg + DXRT | Cisplatinum 100 g m$^{-2}$ d 1<br>Bleomycin 15 g m$^{-2}$ d 3–7 | |
| 25 | M,O | | Ind | DXRT | Cisplatinum 120 g m$^{-2}$ d 4<br>5-FU 600 mg m$^{-2}$ d 1–5<br>Bleomycin i.v. 10 u m$^{-2}$ d 2<br>Methotrexate 120 mg m$^{-2}$ d 2 | |
| 29 | M,O,N,H,L | III-IV | Syn/main | DXRT | Cisplatinum 20 mg m$^{-2}$ i.v. d. 1,2,3<br>Bleomycin 10 g m$^{-2}$ d1,3,5,7 | |
| 30 | O,H,U | III-IV | Ind/main | DXRT ± surg | Cisplatinum 50 mg m$^{-2}$ i.v.<br>Methotrexate 30 mg/m$^{-2}$ i.v.<br>Bleomycin 10 u m$^{-2}$ | Once before and once after DXRT |
| 35 | M,O,H,L | III-IV | IND | Surg + DXRT | Cisplatinum 50 mg m$^{-2}$ i.v.<br>MTX 40 mg/m$^2$ i.v.<br>Bleomycin 15 u m$^{-2}$ i.v.<br>Vincristine 2 mg i.v. | q 21 days |
| 48 | M,O,N,A,G,H | III-IV | IND | DXRT/surg | Cisplatinum 100 g m$^{-2}$<br>5-FU 500 g m$^{-2}$ 1–5 days | |

M, oral cavity; A, nose and sinuses; N, nasopharynx; O, oropharynx; H, hypopharynx; L, larynx; U, unknown parity.

**Table II** Patients' details: methotrexate based regimens

| *Reference* | *Tumour site* | *Tumour stage* | *Type of chemotherapy* | *Local treatment* | *Dose* |
|---|---|---|---|---|---|
| 1 | M,O,A | II-IV | Induction | DXRT | 3–5 mg i.a. d 1–42 |
| 8 | M,A,N,O,H,L, | III-IV | Induction | DXRT | 25 mg i.v. d3,6,9,12,15 |
| 16 | M,O,H,N,A | III-IV | Synchronous | DXRT | 100 mg m$^{-2}$ i.v. d 0 & 14 |
| 23 | A,M,N,O,H,L,U | III-IV | Induction | DXRT | Escalated to 240 mg m$^{-2}$ d 1–5 |
| 32 | M,N,O,H | IV | Induction | DXRT | 50–100 mg kg i.v.<br>Vincristine 0.015 mg kg$^{-1}$ i.v. |
| 33 | M,O,H,L | III-IV | Induction/ maintenance | Surgery | 40 g m$^{-2}$<br>DXRT escalated to 80 mg m$^{-2}$ weekly × 16 |

[a]See Table I.

**Table III** Miscellaneous regimens

| Reference | Tumour site[a] | Tumour stage | Type of chemotherapy | Local treatment | Agent & dose |
|---|---|---|---|---|---|
| 4 | O | II-IV | Syn | DXRT | Bleomycin 15 mg i.m. 2 week$^{-1}$ |
| 12 | M,N,O,H,L | III-IV | Syn/main | DXRT | Bleomycin 5 u i.v. 2 wk$^{-1}$ during DXRT<br>Bleomycin 15 u i.v. weekly × 16 main<br>MTX 25 mg m$^{-1}$ i.v. weeky × 16 main |
| 15,24 | M,O,N,H,L,U | II-IV | Syn | DXRT | 5-FU i.v. 10 mg kg$^{-1}$ daily days 1-3<br>then 5 mg kg$^{-1}$ 3 times weekly |
| 18 | M,A,L,H,N,O | III-IV | Ind/Main | DXRT/Surg | Bleomycin 10 units/t.d.s. d 1-4<br>Cyclophos 200 mg m$^{-2}$ d 1-5<br>Methotrexate 30 g m$^{-2}$ d 1 and 5<br>5-FU 400 mg m$^{-2}$ d 1-5 |
| 20 | M,O,L,N,A,U | I-IV | Syn | DXRT | Hydroxyurea 60 mg kg$^{-1}$ 3 times wkly |
| 40,41 | M,O,L,N,A,H | | Syn | DXRT | Hydroxyurea 80 mg kg$^{-1}$ twice a week |
| 42 | M,L,O,N,H | III-IV | Ind/main | DXRT | Vincristine 1.5 mg m$^{-2}$ i.v. 0 h<br>Methotrexate 75 g m$^{-2}$ 12 h<br>Methotrexate 75 g m$^{2}$ i.v. 15 h<br>Bleomycin 60 mg i.v. 15 h<br>Methotrexate 75 g m$^{-2}$ i.v. 18 h<br>5-FU 350 mg m$^{-2}$ i.v. 18 h<br>Hydroxyurea 3 g m$^{-2}$ 0 h<br>6MP 150 g m$^{-2}$ orally 6 h<br>Cyclophos 500 mg m$^{-2}$ i.v. 12 h |
| 45 | L,H,O,M,N,EAR | III-IV | Ind/main | DXRT | Vinblastine 6 mg m$^{-2}$ 0 h<br>Bleomycin 15 mg i.m. 8 h<br>Methotrexate 40 mg m$^{-2}$ 24 h<br>Cyclophos 400 mg m$^{-2}$ 24 h<br>5-FU 5 mg kg$^{-1}$ 24 h |
| 47 | M,L,O,N,H | III-IV | Ind/main | DXRT + surg | Induction:<br>methotrexate 60 mg/m$^{2}$ 6 hourly<br>d1,5,9<br>Maintenance<br>cisplatinum 40 mg m$^{-2}$)<br>adriamycin 40 mg m$^{-2}$) 3 wks × 4 |
| 49 | M,O,N,H,L,A | II-IV | Synch | DXRT | Bleomycin total dose 100 mg i.v. |

[a]See Table I.

*Response rates*

These apply only to induction trials. The response rates are summarised where they have been reported, and the survival in these trials analysed.

*Meta-analysis*

The appropriate data for survival were extracted from the papers quoted and were subject to meta-analysis (Peto, 1987). The mathematical basis of this technique is shown in Appendix 2.

The death rate (number of deaths/total number) used for these analyses was that up to 2 years where these data were available. In some reports, information about the outcome of each patient is provided but, in general, this was not so. In most of the papers, any losses to follow-up during the first 2 years could not be taken into acount and the quoted death rates may, therefore, be slightly too low.

This small bias applies equally across treatment groups, however, and should not affect the significance of the survival comparison. In those trials in which the number of deaths by 2 years was not given, the number of deaths (rounded to the nearest integar) was calculated as (1 − S) × (total number of patients), where S is the survival rate at 2 years estimated from the relevant survival curve. In five series data were available at only one point in time, and not at two years. The data used were then the death rate at 1 year (Martin *et al.*, 1986; Stolwijk *et al.*, 1983), 18 months (Petrovich *et al.*, 1981), 5 years (Stefani & Chung, 1980) and at the time of writing (Gollin *et al.*, 1972). These problems mean that the meta-analysis results are not exact. Nevertheless, they are good enough to indicate any trends.

A meta-analysis is provided of the pooled results of all the trials, together with subgroup analyses for the main groups of chemotherapy regimens, for the different timing of administration (induction, synchronous and maintenance), for various sites and for single agents versus combinations.

The odds ratios with approximate 95% confidence intervals for these different forms of treatment were also calculated using the formulae shown in Appendix 3.

*Site of failure*

The concept is gaining ground that adjuvant chemotherapy might be useful in reducing the proportion of patients who require salvage surgery for failed radiotherapy. Also some believe that it might increase the rate of distant metastases (Vermund *et al.*, 1985), and some that it might reduce it (Schuller *et al.*, 1988). Therefore data for site of failure were extracted when they were available.

*Toxicity*

All papers were reviewed for reports of toxicity, in particular deaths and the use of a grading system as recommended by the WHO (Miller *et al.*, 1981).

*Cost*

The cost of induction and maintenance regimens was calculated based on the cost of a bed per day in an English teaching hospital in 1987, which was £152.78 (Stell, 1987). The length of stay was that given in each article, plus an arbitary 3 days which is usually required for assessment, and for recovery from the effects of chemotherapy. The cost of drugs was ignored as this is relatively small; furthermore the cost of synchronous chemotherapy was not calculated as it usually does not require an extra stay in hospital, and its cost is, therefore, minimal.

### Results

### *Design of the trial*

*Power of the trial* The minimal difference in survival which could be detected by each trial ranged from 11 to 51% with a median of 25%.

*Randomisation* All the trials reviewed were randomised, but many did not describe the randomisation process. Four trials did not specify whether eligible patients were excluded from randomisation. Twelve authors described exclusion of eligible patients before randomisation, but only five authors (Ervin *et al.*, 1987; Fu *et al.*, 1987; Holoye *et al.*, 1985; Schuller *et al.*, 1988; Toohill *et al.*, 1987) give details of how many eligible patients were excluded. These latter five trials contained a total of 648 patients, 134 (20.7%) of eligible patients were excluded before randomisation. In four trials there were no exclusions from randomisation, but some treated patients were excluded from analysis (v.i.). Only five adhered to the policy of 'intention to treat' (Gupta *et al.*, 1987; Pearlman *et al.*, 1985; Stell *et al.*, 1983; Stolwijk *et al.*, 1983; Vermund *et al.*, 1985), i.e. all eligible patients were randomised and included in the analysis, whether treated or not.

### *Analysis of survival*

*Exclusion of treated patients* Nine reports excluded some treated patients from the final analysis (Cachin *et al.*, 1977; Fazekas *et al.*, 1980; Fu *et al.*, 1987; Head and Neck Contracts Program, 1987; Hussey & Abrams, 1975; Lo *et al.*, 1976; Martin *et al.*, 1986; Stefani & Chung, 1980; Taylor *et al.*, 1985). Out of a total of 2012 patients in these nine trials 183 patients were excluded from the analysis (9.1%).

*Method of calculation of survival* Eleven authors analysed cancer deaths alone, three reported total mortality, one did not state how survival was calculated and seven authors gave full details of the fate of all patients at a specific interval. From these latter seven it is possible to calculate the difference between cancer survival and crude survival (Holoye *et al.*, 1985; Knowlton *et al.*, 1975; Pearlman *et al.*, 1985; Petrovich *et al.*, 1981; Taylor *et al.*, 1985; Toohill *et al.*, 1987; Vermund *et al.*, 1985) (Table IV).

Chemotherapy increased the total mortality by 5.0% whereas the difference in cancer death rates was 2.4%. Thus, an analysis restricted to cancer deaths underestimated the death rate by 2.6%, probably because deaths due to toxicity were not included in the analysis.

### *Response rates*

Response rates using WHO criteria were only quoted in four of the induction studies. The results (Table V) show that a response rate of 46.9% equated with an increase in death rate of 6.4%, although this change was not significant.

### *Meta-analysis*

A total of 3,683 patients were eligible and of these, 3,398 patients were randomised. The death rate was 885/1,560 (56.7%) in the control arms, and 969/1724 (56.2%) in the treated arms, an improvement of survival of 0.5% in the chemotherapy arms. Meta-analysis showed that the sum of $(O-E)$ over all trials was $-4.29$ and its variance 201.39. This difference is not significant ($z=-0.30$). The odds ratio was 1.02 and its 95% confidence interval 0.89–1.17.

The subgroup analysis was carried out for the different agents, irrespective of whether they were used for induciton, maintenance, etc. (Tables VI to IX). This showed that the VBM regimen significantly increased the death rate from cancer, whereas all the other regimens were ineffective.

Subgroup analysis for the timing of chemotherapy (irrespective of the agents used) is shown in Table X. Induction chemotherapy, synchronous chemotherapy and induction/maintenance were completely ineffetive, whereas synchronous/maintenance therapy significantly reduced deaths from cancer ($P<0.05$).

A subgroup analysis for site (irrespective of agents and timing of use) is given in Table XI. No significant differences were found.

Finally, subgroup analysis of the number of agents (irrespective of agent and timing of use) showed that neither single agents nor multiple agents produced a significant reduction of the death rate.

### *Site of failure*

Data about the site of failure were given in 10 papers. The results are summarised in Tables XIII and XIV. The failure rate from locoregional recurrence was 6.7% less in the treated groups, and this difference was highly significant ($P<0.01$). The failure rate for distant metastases was 2.0% lower in the treated group, but this was not significant.

### *Toxicity*

Toxicity was reported fully and graded in only three papers (Ervin *et al.*, 1987; Fu *et al.*, 1987; Schuller *et al.*, 1988). Nine papers gave details of toxicity, with no grading scheme, eight papers dismissed it in a few words, e.g. 'mild and transient' (Arcangeli *et al.*, 1983), and four did not mention it at all. No paper used the scheme recommended by the WHO (Miller *et al.*, 1981).

Deaths from toxicity were mentioned in 10 series (Ervin *et*

**Table IV** Difference between total and cancer mortality

| | *Control arm* | *Treated arm* | *(O-E)* | *Variance* | *Z* | *P* |
|---|---|---|---|---|---|---|
| Total mortality | 158/311 | 172/308 | | | 1.26 | n.s. |
| | (50.8%) | (55.8%) | 7.80 | 38.58 | | |
| Cancer mortality | 157/311 | 163/308 | | | | |
| | (50.5%) | (52.9%) | 3.78 | 38.70 | 0.61 | n.s. |

**Table V** Response rates and survival

| | *Response* | *Deaths* | | | | | |
|---|---|---|---|---|---|---|---|
| *Reference* | *rate* | *Control arm* | *Treated arm* | *O-E* | *Var* | *Z* | *P* |
| 17 | 104/282 | 67/152 | 62/140 | 0.15 | 18.03 | 0.04 | n.s. |
| 25 | 18/28 | 9/27 | 14/28 | 2.29 | 3.41 | 1.24 | n.s. |
| 32 | 8/12 | 10/11 | 10/12 | −0.43 | 0.68 | −0.52 | n.s. |
| 35 | 56/75 | 34/76 | 49/82 | 5.92 | 9.90 | 1.88 | n.s. |
| Total | 186/397 | 120/266 | 135/262 | 8.47 | 33.02 | 1.47 | n.s. |
| | (46.9%) | (45.1%) | (51.5%) | | | | |

**Table VI** Meta-analysis: cisplatinum regimens

| | Deaths | | | | | |
|---|---|---|---|---|---|---|
| *Reference* | *Control arm* | *Chemotherapy arm* | *O-E* | *Variance* | *Z* | *P* |
| 7 | 7/20 | 3/26 | −2.65 | 1.97 | −1.89 | n.s. |
| 17 | 67/152 | Ind 62/140 | 0.15 | 18.03 | 0.04 | n.s. |
| | | Main 59/151 | −3.79 | 18.46 | −0.88 | n.s. |
| 25 | 9/27 | 14/28 | 2.29 | 3.41 | 1.24 | n.s. |
| 29 | 13/13 | 15/23 | −2.89 | 1.48 | −2.38 | <0.025 |
| 30 | 18/28 | 15/31 | −2.34 | 3.69 | 1.22 | n.s. |
| 35 | 34/76 | 49/82 | 5.92 | 9.90 | 1.88 | n.s |
| 48 | 11/33 | 12/27 | 1.65 | 3.57 | 0.87 | n.s. |
| Total | 159/349 (45.6%) | 229/508 (45.1%) | −0.99 | 51.32 | −0.14 | n.s. |
| | | Odds ratio 1.02 (0.78−1.34) | | | | |

**Table VII** Meta-analysis: methotrexate regimens

| | Deaths | | | | | |
|---|---|---|---|---|---|---|
| *Reference* | *Control arm* | *Chemotherapy* | *O-E* | *Variance* | *Z* | *P* |
| 1 | 35/70 | 26/72 | −4.93 | 8.76 | −1.67 | n.s. |
| 8 | 225/326 | 223/312 | 3.92 | 33.79 | 0.68 | n.s. |
| 16 | 83/157 | 72/156 | −5.25 | 19.62 | −1.19 | n.s. |
| 23 | 34/48 | 41/48 | 3.50 | 4.14 | 1.72 | n.s. |
| 32 | 10/11 | 10/12 | −0.43 | 0.68 | −0.52 | n.s. |
| 33 | 10/27 | 9/28 | −0.67 | 3.17 | −0.38 | n.s. |
| Total | 397/639 (62.1%) | 381/628 (60.7%) | −4.62 | 75.12 | −0.53 | n.s. |
| | | Odds ratio 1.06 (0.85−1.33) | | | | |

**Table VIII** Meta-analysis: VBM regimens

| | Deaths | | | | | |
|---|---|---|---|---|---|---|
| *Reference* | *Control arm* | *Treated arm* | *(O-E)* | *Variance* | *Z* | *P* |
| 42 | 1839 | 34/47 | 5.58 | 5.16 | 2.46 | <0.025 |
| 45 | 8/35 | 14/33 | 3.32 | 3.77 | 1.71 | n.s. |
| Total | 26/74 (35.1%) | 48/80 (60.0%) | 9.56 | 9.66 | 3.08 | <0.01 |
| | Odds ratio 0.36 (0.19−0.69) | | | | | |
| *Bleomycin regimens* | | | | | | |
| 4 | 48/87 | 57/99 | 1.11 | 11.45 | 0.33 | n.s. |
| 12 | 33/51 | 22/45 | −3.78 | 5.91 | −1.55 | n.s. |
| 49 | 40/111 | 48/111 | 4.0 | 13.34 | 1.10 | n.s. |
| Total | 121/249 (48.6%) | 127/255 (49.8%) | 1.52 | 31.55 | 0.27 | n.s. |
| | Odds ratio 0.95 (0.67−1.35) | | | | | |
| *5-FU regimen* | | | | | | |
| 15,24 | 64/79 (81.0%) | 52/76 (68.4%) | −4.88 | 7.34 | −1.80 | n.s. |
| | Odds ratio 1.97 (0.94−4.13) | | | | | |

**Table IX** Meta-analysis: miscellaneous regimens

| | Deaths | | | | | |
|---|---|---|---|---|---|---|
| *Author* | *Control arm* | *Chemotherapy arm* | *(O-E)* | *Variance* | *Z* | *P* |
| 18 | 23/39 | 25/38 | 1.31 | 4.58 | 0.61 | n.s. |
| 20 | 11/15 | 16/23 | −0.34 | 1.92 | −0.25 | n.s. |
| 40,41 | 62/75 | 70/75 | 4.00 | 3.99 | 2.00 | <0.05 |
| 47 | 22/41 | 21/41 | −0.50 | 5.18 | −0.22 | n.s. |
| Total | 118/170 (69.4%) | 132/177 (74.6%) | 4.48 | 17.51 | 1.07 | n.s. |
| | Odds ratio 0.77 (0.48−1.24) | | | | | |

*al.*, 1987; Fazekas *et al.*, 1980; Fu *et al.*, 1987; Holoye *et al.*, 1985; Knowlton *et al.*, 1975; Nissenbaum *et al.*, 1984; Pearlman *et al.*, 1985; Schuller *et al.*, 1988; Stell *et al.*, 1983; Stolwijk *et al.*, 1983). One of these did not record the number of deaths due to toxicity, but stated that serious complications, including death, occurred in 'less than 10% of all patients' (Knowlton *et al.*, 1975). In the remaining nine reports, the total mortality from chemotherapy was 41/627, i.e. 6.5%. The death rate in these nine trials totalled 379/627 in the control arm and 400/637 in the treated arm. Thus, in these nine trials the death rate was 2.4% *higher* in the treated arms, to which must be added a further 6.5% increase in deaths due to toxicity, making a total *reduction* in survival of 8.9%. Eight of these 10 trials were of induction chemotherapy, and two were of synchronous chemotherapy.

### *Cost*

One paper (Pearlman *et al.*, 1985) mentioned the issue of cost but did not measure it. No other paper mentioned this aspect. None recorded the number of days in hospital. However, a very crude calculation based on the number and length of the courses shows that the median number of days was 22, with a range of 3−50. Thus, the cost ranged from £458 to £7,639, the median being £3,361.

### Discussion

Given that the overall survival of squamous carcinoma of the head and neck has not improved in a generation (Stell *et al.*, 1986) it is very unlikely that a major improvement in survival is to be expected from manipulations of the various forms of treatment currently available. Real improvement in survival of 5% would be very worthwhile and is realistically all that might be achieved in the present circumstances; a 10% improvement is just conceivable, but it is unreasonable to expect any improvement beyond this with currently available forms of treatment.

This overview shows that no trial yet mounted is big enough to detect the probable degree of improvement from chemotherapy, that is between 5 and 10%. The least difference which could be reliably detected by these trials was 11%, and the median was 25%. Any new form of treatment which produces an improvement in survival as great as 25% would be adopted rapidly, without the necessity for a trial.

Only about a quarter of these trials adhered to the policy of 'intention to treat', and many excluded some eligible patients from randomisation. Where details were given, it emerged that over a third of eligible patients were excluded before randomisation. Furthermore, 10% of treated patients

**Table X** Meta-analysis: type of chemotherapy

| *Reference* | *Sum (O-E)* | *Sum (var)* | *Z* | *P* | *Odds ratio (95% CI)* |
|---|---|---|---|---|---|
| *Induction* | | | | | |
| 1,8,23,25,32 35,48 | 11.63 | 68.69 | 1.40 | n.s. | 0.84 (0.67–1.07) |
| *Synchronous* | | | | | |
| 4,15,16,20,24, 40,41,49 | −1.18 | 64.60 | −0.15 | n.s. | 1.02 (0.80–1.30) |
| *Induction/maintenance* | | | | | |
| 7,17,18,30,33, 42,45,47 | 0.39 | 55.20 | −0.05 | n.s. | 1.01 (0.77–1.31) |
| *Synchronous/maintenance* | | | | | |
| 12,29 | −5.76 | 7.75 | −2.07 | <0.05 | 2.14 (1.04–4.42) |

**Table XI** Meta-analysis: site

| *Reference* | *Sum (O−E)* | *Sum (var)* | *Z* | *P* | *Odds ratio (95% CI)* |
|---|---|---|---|---|---|
| *Mouth* | | | | | |
| 1,8,16, 24 | −9.0 | 21.60 | −1.94 | n.s. | 1.52 (0.99–2.33) |
| *Oropharynx* | | | | | |
| 1,4,8,16 24,32 | −7.93 | 42.24 | −1.22 | n.s. | 1.21 (0.89–1.63) |
| *Larynx & hypopharynx* | | | | | |
| 8,15,16 | −3.16 | −0.80 | n.s. | 1.22 | (0.75–2.01) |

**Table XII** Meta-analysis: number of agents

| *Reference* | *Sum (O−E)* | *Sum (var)* | *Z* | *P* | *Odds ratio (95% CI)* |
|---|---|---|---|---|---|
| *Single agent* | | | | | |
| 1,4,8,15, 16,20, 23,24,33, 40,41,49 | −0.54 | 118.20 | −0.05 | n.s. | 1.00 (0.84–1.20) |
| *Multiple agents* | | | | | |
| 7,12,17,18, 25,29,30,32,35, 42,45,47,48 | 2.27 | 79.35 | 0.25 | n.s. | 0.97 (0.78–1.21) |

**Table XIII** Meta-analysis: locoregional failure

| *Reference* | *Control arm* | *Treated arm* | *(O−E)* | *Var* | *Z* | *P* |
|---|---|---|---|---|---|---|
| 1 | 44/70 | 32/72 | −6.54 | 8.89 | −2.19 | <0.05 |
| 8 | 75/173 | 69/164 | −1.08 | 20.66 | −0.24 | n.s. |
| 16 | 72/156 | 53/156 | −9.50 | 18.79 | −2.19 | <0.05 |
| 17 | 36/144 | 35/135) | | | | |
| | | 31/132) | −0.26 | 17.50 | −0.06 | n.s. |
| 18 | 16/39 | 24/38 | 4.26 | 4.87 | 1.93 | n.s. |
| 20 | 10/16 | 15/24 | 0.00 | 2.31 | 0.00 | n.s. |
| 23 | 33/48 | 37/48 | +2.00 | 4.79 | 0.91 | n.s. |
| 24 | 38/56 | 27/56 | −5.50 | 6.88 | −2.10 | <0.05 |
| 48 | 10/33 | 12/27 | 2.10 | 3.51 | 1.12 | n.s. |
| 49 | 70/111 | 61/111 | −4.5 | 13.48 | −1.22 | n.s. |
| Total | 404/846 (47.8%) | 396/963 (41.1%) | −29.87 | 111.15 | −2.83 | <0..01 |

Odds ratio 1.31 (1.09–1.58).

**Table XIV** Meta-analysis: distant metastases

| *Reference* | *Control arm* | *Treated arm* | *(O−E)* | *Var* | *Z* | *P* |
|---|---|---|---|---|---|---|
| 1 | 13/70 | 12/72 | −0.68 | 5.19 | −0.30 | n.s. |
| 8 | 27/173 | 16/164 | −4.93 | 9.40 | −1.61 | n.s. |
| 17 | 27/144 | 26/135) | −4.23 | 12.49 | −1.20 | n.s. |
| | | 12/132) | | | | |
| 18 | 5/39 | 1/38 | −1.96 | 1.40 | −1.66 | n.s. |
| 23 | 11/48 | 6/48 | −2.50 | 3.53 | −1.33 | n.s. |
| 24 | 6/56 | 5/56 | −0.50 | 2.50 | −0.32 | n.s. |
| 48 | 2/33 | 1/27 | −0.35 | 0.72 | −0.41 | n.s. |
| 49 | 13/111 | 26/111 | 6.50 | 8.07 | 2.29 | <0.025 |
| Total | 104/674 (15.4%) | 105/783 (13.4%) | −7.32 | 44.54 | −1.10 | n.s. |

Odds ratio 1.18 (0.88–1.56).

were excluded from analysis and this must be an enormous source of bias because it is highly likely that excluded patients do badly. A further source of bias of any review of published trials is the fact that small trials and negative trials are unlikely to be reported (publication bias). Finally, most trials did not report the actual mechanism of randomisation, and it may be that some used systems, such as randomising by the date of birth, which allow the clinician to know beforehand the treatment arm to which a patient would be allocated.

Most of the trials relied on analysis of cancer deaths rather than total mortality. Although chemotherapy does not affect the rate of intercurrent deaths, nor the survival of patients lost to follow-up, calculation should nonetheless be based on total mortality because deaths from toxicity may be counted as intercurrent deaths, thus giving a false sense of the overall picture. The analysis of cancer mortality rather than crude survival in the series reviewed here inflated the survival rate of the chemotherapy group by 2.6%, indicating that some deaths due to toxicity were counted as intercurrent deaths. The analysis of deaths due to other causes is a serious problem in trials in which they contribute a substantial proportion of total mortality. However, the risk of death from other causes is low during the first few years after diagnosis and, for trials of advanced cancer, the simplest and most reliable analysis is that based on total mortality.

Meta-analysis showed that, overall, the chemotherapy arms fared slightly better than the control arms, the difference in cancer mortality being 0.5%. However, analysis of the seven trials which gave full details and were, therefore, the most reliable showed an increase of 5.0% in mortality.

Subgroup analysis suggests that cisplatinum, methotrexate, bleomycin, 5-FU and miscellaneous regimens were ineffective, whereas VBM produces a significant reduction in survival. Subgroup analysis also suggests that synchronous/ maintenance therapy is probably effective (although this result is based on only two trials), whereas induction, induction/maintenance and synchronous therapy are not. Single agents are not better than combinations. Finally, adjuvant therapy does not benefit tumours of any particular site.

The available data for site of failure suggest that chemotherapy reduces the rate of locoregional failure, thus improving quality of survival. There is no evidence that it either increases or decreases the distant metastatic rate.

Although many authors dismiss toxicity lightly, it is clear form the few available data from the death rate of 6.5% form toxicity that this conclusion is not justified.

The figures for cost are very aproximate and do not include drug costs. They also relate only to induction and maintenance regimens, since synchronous regimens usually do not prolong stay in hospital. It has been shown that the maximum cost per patient, per year of life saved, that the UK National Health Service can sustain is £17,000 at 1987 prices (Stell, 1987). Induction or maintenance chemotherapy would thus need to increase survival by 20–25% to be affordable. As they increase survival by 5% at most, they are not affordable.

This review has many sources of bias, some of which have already been referred to, but the main source of inaccuracy is the difficulty of extracting complete data from the various reports. In particular the survival at 2 years often had to be read off a very small graph; although the number of patients admitted to the trial was always given, the number at risk at this or any other time interval was often not given. For some of the studies an alternative end-point had to be used, e.g. 5 year survival. This was not ideal but the sum of the observed and expected numbers of deaths from these analyses still provides a valid and reasonably efficient global test of whether there is a difference in survival.

Moreover, the subgroup analyses should be treated with great caution. Subgroup analysis should only be carried out if the following criteria are satisfied: (a) the analysis was intended at the start of the trial; (b) the analysis is not liable to bias; (c) the analysis is biologically plausible; (d) the result of the trial is significant overall (Bulpitt, 1988). Therefore, Peto (1987) advises reporting subgroup analyses but treating them with grave suspicion. However, they may be useful pointers to further trials.

The authors wish to make a strong plea to both authors and editors of journals that reports of cancer clinical trials should include the following:

1. Calculation of sample size and of the minimal difference likely to be detected by the trial (the power of the study).
2. Details of the fate of eligible patients excluded form randomisation.
3. Details of the fate of treated patients excluded from the analysis.
4. Both total and cancer mortality.
5. A full table of results at 2 and 5 years.
6. Toxicity, using the WHO scale.
7. Deaths from toxicity.
8. Cost.

### Appendix 1

The formula for calculating the minimal number of events needed to detect a given difference in an individual was adapted from the following formula given by George (1984).

$$n = \frac{(Z_{a/2} + Z_b)^2}{2\,(\text{arc sin}\ \sqrt{r_1} - \text{arc sin}\ \sqrt{r_2})^2}$$

where $r_1$ = 'cure' rate in control group; $r_2$ = 'cure' rate in treated group; $Z_{a/2}$ = normal deviate for type 1 error; $Z_b$ = normal deviate for Type II error; $n$ = number in each arm.

### Appendix 2

The meta-analysis is that described by Peto (1987).

The expected number of deaths in the treated arm is calculated from the overall result, and this number is subtracted from the observed number of deaths. The resulting quantity (O – E) will be negative if the treatment is effective, that is the number of deaths is reduced. The standard deviation of $(O-E)$ can be calculated so that the values for $(O-E)$ and its variance can be summed over several trials. The sum of $(O-E)$ is divided by its standard deviation (the square root of the summed variances) to test for a significant effect in the trials as a whole.

The survival data for a two arm trial can be displayed as shown below:

| | *Treatment 1* | *Treatment 2* |
|---|---|---|
| Died | $r_1$ | $r_2$ |
| Survived | $n_1 - r_1$ | $n_2 - r_2$ |
| | $n_1$ | $n_2$ |

where $r_1$ = no. of deaths in group 1; $r_2$ = no. of deaths in group 2; $n_1$ = total no. in group 1; $n_2$ = total no. in group 2.

The parameter $O-E$ (observed – expected number of deaths) for the treated group is calculated as follows:

$$O - E = (n_2)\,\frac{(r_1 + r_2)}{N} - r_2$$

The variance $V$, of $O - E$

$$= n_1 P\ Q\ (N - n_1)/(N - 1)$$

where $N = n_1 + n_2$; $P = (r_1 + r_2)/N$; $Q = 1 - P$.

The significance of $(O-E)$ is tested by dividing it by the square root of its variance, and referring the result to tables of the normal distribution.

### Appendix 3. Odds ratio

If the results are displayed in a table as shown in Appendix 2, the proportion in each cell can be readily calculated as follows:

$$p_{11} = r_1/N$$

$$p_{12} = r_2/N$$

$$p_{21} = (n_1 - r_1)/N$$

$$p_{22} = (n_2 - r_2)/N$$

and can be displayed as follows:

| | *Control arm* | *'Treated' arm* |
|---|---|---|
| Dead | $p_{11}$ | $p_{12}$ |
| Alive | $p_{21}$ | $p_{22}$ |

The odds ratio, $R$, is then calculated as follows (Fleiss, 1981):

$$R = (p_{11} \times p_{22})/(p_{12} \times p_{21})$$

The standard deviation is given as follows:

$$\text{s.d. } (\log R) = \frac{1}{\sqrt{N}} \left( \frac{1}{p_{11}} + \frac{1}{p_{12}} + \frac{1}{p_{21}} + \frac{1}{p_{22}} \right)^{0.5}$$

Approximate 95% confidence intervals are given as follows:

$$\exp\ (\log R \pm 1.96 \text{ s.d. } (\log R))$$

The authors are very grateful to the following who read the manuscript and made many valuable suggestions: Dr Marc Buyse, Assistant Director, EORTC Data Centre, Boulevard de Waterloo 125, 1000 Brussels, Belgium; Professor Julian Peto, Section of Epidemiology, Institute of Cancer Research, Block D, 15 Cotswold Road, Belmont, Surrey SM2 5NG, United Kingdom; Dr S.G. Taylor IV, Department of Internal Medicine, Section of Medical Oncology, Rush Medical College, 1725 West Harrison Street, Chicago, Il 60612, USA. The authors are also grateful to Mrs J. Deeprose and Mrs B. Cowley for the typing.

### References

ARCANGELI, G., NERVI, C., RIGHINI, R.E., CRETON, R., MIRRI, M.A. & GUERRA, A. (1983). Combined radiation and drugs: the effect of intra-arterial chemotherapy followed by radiotherapy in head and neck cancer. *Radiother. Oncol.*, **1**, 101.

BAKOWSKI, M.T., MACDONALD, E., MOULD, R.F. & 8 others (1978). Double blind controlled clinical trial of radiation plus razoxane (ICRF 159) versus radiation plus placebo in the treatment of head and neck cancer. *J. Radiat. Oncol. Biol. Phys.*, **4**, 115.

BULPITT, C.J. (1988). Subgroup analysis. *Lancet*, **i**, 31.

CACHIN, Y., JORTRAY, A., SANCHOS, H. & 4 others (1977). Preliminary results of a randomised E.O.R.T.C. study comparing radiotherapy and concomitant Bleomycin, to radiotherapy alone in epidermoid carcinomas of the oropharynx. *Eur. J. Cancer*, **13**, 1389.

CACHIN, Y. (1982). Adjuvant chemotherapy in head and neck carcinoma. *Clin. Otolaryngol.*, **3**, 121.

CHANG, T.M. (1988). Induction chemotherapy for advanced head and neck cancers: a literature review. *Head Neck Surg.*, **10**, 150.

ERVIN, T.J., CLARK, J.R., WEICHSELBAUM, R.R. & 9 others (1987). An analysis of induction and adjuvant chemotherapy in the multidisciplinary treatment of squamous cell carcinoma of the head and neck. *J. Clin. Oncol.*, **5**, 10.

FAZEKAS, J.T., SOMMER, C. & KRAMER, S. (1980). Adjuvant intravenous Methotrexate or definitive radiotherapy alone for advanced squamous cancers of the oral cavity, oropharynx, supraglottic larynx or hypopharynx. *Int. J. Radiat. Oncol. Biol. Phys.*, **6**, 533.

FLEISS, J.L. (1981). *Statistical Methods for Rates and Proportions*, 2nd edn. John Wiley: New York.

FLETCHER, G.H., SUIT, H.D., HOWE, C.D., SAMUEL, S.M., JESSE, R.H. & VILLAREAL, R.U. (1963). Clinical method of testing radiation-sensitizing agents in squamous cell carcrinoma. *Cancer*, **16**, 355.

FORESTIERE, A.A. (1986). Review: management of advanced stage squamous cell carcinoma of the head and neck. *Ann. J. Med. Sci.*, **291**, 405.

FU, K.K., PHILLIPS, T.L., SILVERBERG, I.J. & 7 others (1987). Combined radiotherapy and chemotherapy with Bleomycin and Methotrexate for advanced inoperable head and neck cancer: update of a Northern California Oncology Group Randomized Trial. *J. Clin. Oncol.*, **5**, 1410.

GEORGE, S.L. (1984). The required size and length of a phase III clinical trial. In *Cancer Clinical Trials–Methods and Practice*, Buyse, M.E., Staquet, M.J. & Sylvester, R.J. (eds) p.289 Oxford University Press: Oxford.

GLICK, J.H. & TAYLOR, S.G. IV (1981). Integration of chemotherapy into a combined modality treatment plan for head and neck cancer: a review. *Int. J. Radiat. Oncol. Biol. Phys.*, **7**, 229.

GOLLIN, F.F., ANSFIELD, F.J., BRANDENBURG, J.H., RAMIREZ, G. & VERMUND, H. (1972). Combined therapy in advanced head and neck cancer: a randomized study. *Am. J. Roentgenol.*, **114**, 83.

GUPTA, N.K., POINTON, R.C.S. & WILKINSON, P.M. (1987). A randomised clinical trial to contrast radiotherapy and Methotrexate given synchonously in head and neck cancer. *Clin. Radiol.*, **38**, 575.

HEAD AND NECK CONTRACTS PROGRAM (1987). Adjuvant chemotherapy for advanced head and neck squamous carcinoma. *Cancer*, **60**, 301.

HOLOYE, P.Y., GROSSMAN, T.W., TOOHILL, R.J. & 7 others (1985). Randomized study of adjuvant chemotherapy for head and neck cancer. *Otolaryngol Head and Neck Surg.*, **93**, 712.

HONG, W.K. (1988). Editorial. Induction chemotherapy for advanced head and neck cancer. *Head Neck Surg.*, **10**, 147.

HUSSEY, D.H. & ABRAMS, J.P. (1975). Combined therapy in advanced head and neck cancer: hydroxyurea and radiotherapy. *Prog. Clin. Cancer*, **6**, 79.

JOHNSON, J.T., MYERS, E.N., STRODES, C.H. & 5 others (1985). Maintenance chemothearapy for high-risk patients. *Arch. Otolaryngol*, **111**, 727.

KAPSTAD, B., BANG, G., RENNAES, S. & DAHLER, A. (1978). Combined properative treatment with cobalt and bleomycin in patients with head and neck carcinoma–a controlled clinical study. *J. Radiat. Oncol. Biol. Phys.*, **4**, 85.

KNOWLTON, A.H., PERCARPIO, B., BOBROW, S. & FISCHER, J.J. (1975). Methotrexate and radiation therapy in the treatment of advanced head and neck tumors. *Ther. Radiol.*, **116**, 709.

LO, T.C., WILEY, A.L., ANSFIELD, F.J. & 6 others (1976). Combined radiation therapy and 5-fluorouracil for advanced squamous cell carcioma of the oral cavity an oropharynx. A randomised study. *Am. J. Roentgenol.*, **126**, 229.

MARTIN, M., MAZERON, J.J., GLAUBIGER, D. & 9 others (1986). Neo-adjuvant polychemotherapy of head and neck cancer: preliminary results of a randomised study. *Proc. ASCO*, **5**, 141.

MEAD, G.M. & JOCOBS. C. (1982). Changing role of chemotherapy in treatment of head and neck cancer. *Am. J. Med.*, **73**, 582.

MILLER, A.B., HOOGSTRATEN, B., STAQUET, M. & WINKLER, A. (1981). Reporting results of cancer treatment. *Cancer*, **47**, 207.

MORTON, R.P., RUGMAN, F., DORMAN, E.B. & 5 others (1985). Cisplatinum and Bleomycin for advanced or recurrent squamose cell carcinoma of the head and neck: a randomised factorial phase III controlled trial. *Cancer Chemother. Pharmacol.*, **15**, 283.

NISSENBAUM, M., BROWDE, A., BEZWODA, W.R., DE MOOR, N.G. & DERMAN, D.P. (1984). Treatment of advanced head and neck cancer: multiple daily dose fractionated radiation therapy and sequential multimodal treatment approach. *Med. Pediatr. Oncol.*, **12**, 204.

PEARLMAN, N.W., JOHNSON, F.B., BRAUN, T.J. & 6 others (1985). A prospective study of preoperative chemotherapy and spit-course irradiation for locally advanced or recurrent oral/pharyngeal squamous carcinoma. *Am. J. Clin. Oncol.*, **8**, 490.

PETO, R. (1987). Why do we need systematic overviews of randomized trials? *Stat. Med.*, **6**, 233.

PETROVICH, Z., BLOCK, J., KUISK, H. & 4 others (1981). A randomised comparison of radiotherapy with a radiotherapy-chemotherapy combination in Stage IV carcinoma of the head and neck. *Cancer*, **47**, 2259.

RENTSCHLER, R.E., WILBUR, D.W., PETTI, G.H. & 4 others (1987). Adjuvant Methotrexate escalated to toxicity for resectable stage III and IV squamous head and neck carcinomas–a prospective randomized study. *J. Clin. Oncol.*, **5**, 278.

RICHARDS, G.J. & CHAMBERS, G. (1969). Hydroxyurea: a radiosensitizer in the treatment of neoplasms of the head and neck. *Am. J. Roentgenol.*, **105**, 555.

SCHULLER, D.E., METCH, B. MATTOX, D., STEIN, D.W. & MCCRACKEN, J.D. (1988). Preoperative chemotherapy in advanced resectable and head and neck cancer: final report of the Southwest Oncology Group. *Laryngoscope*, **98**, 1205.

SCHULLER, D.E., STEIN, D.W. & METCH, B. (1989). Analysis of treatment failure patterns. *Arch. Otolaryngol. Head Neck Surg.*, **115**, 834.

SECOG. Cancer Research Campaign Clinical Trial Centre (1986). A randomized trial of combined multidrug chemotherapy and radiotherapy in advanced squamous cell carcinoma of the head and neck. *Eur. J. Surg. Oncol.*, **12**, 289.

SECOND INTERNATIONAL STUDY OF INFARCT SURVIVAL COLLABORATIVE GROUP (1988). Randomised trial of intravenous Streptokinase, oral aspirin, both, or neither among 17 187 cases of suspected acute myocardial infactions: ISIS 2. *Lancet*, **ii**, 349.

SHANTA, V. & KRISHNIAMURTHI, S. (1980). Combined Bleomycin and radiotherapy in oral cancer. *Clin. Radiol.*, **31**, 617.

STEFANI, A., EELLS, R.W. & ABBATE, J. (1971). Hydroxyurea and radiotherapy in head and neck cancer. *Radiology*, **101**, 391.

STEFANI, A. & CHUNG, T.S. (1980). Hydroxyurea and radiotherapy in head and neck cancer–long term results of a double blind randomised prospective study. *Radiat. Oncol. Biol. Phys.*, **6**, 1398.

STELL, P.M., DALBY, J.E., STRICKLAND, R., FRASER, J.G., BRADLEY, P.J. & FLOOD, L.M. (1983). Sequential chemotherapy and radiotherapy in advanced head and neck cancer. *Clin. Radiol.*, **34**, 463.

STELL, P.M. & MCCORMICK, M.S. (1986). Cancer of the head and neck: are we doing any better. *Eur. J. Surg. Oncol.*, **12**, 94.

STELL, P.M. (1987). Can we afford to treat head and neck cancer? *Clin. Otolaryngol.*, **12**, 321.

STOLWIJK, C., WAGENER, D.J., VAN DEN BROEK, P., LEVENDAG, P.C., KAZEMI, I. & BRUASET, I. (1983). Randomized adjuvant chemotherapy trial for advanced head and neck cancer. *Netherlands J. Med.*, **28**, 347.

TANNOCK, I.F. & BROWMAN, G. (1986). Lack of evidence for a role of chemotherapy in the routine management of locally advanced head and neck cancer. *J. Clin. Oncol.*, **4**, 1121.

TAYLOR, S.G., APPLEBAUM, E., SHOWEL, J.L. & 5 others (1985). A ranodmized trial of adjuvant chemotherapy in head and neck cancer. *J. Clin. Oncol.*, **3**, 672.

TOOHILL, R.J., ANDERSON, T., BYHARDT, R.W. & 10 others (1987). Cisplatin and fluorouracil as neoadjuvant therapy in head and neck cancer. *Arch. Otolaryngol. Head and Neck Surg.*, **113**, 758.

VERMUND, H., KAALHUS, O., WINTHER, F., TRAUSIO, J., THORUD, E. & HARANG, R. (1985). Bleomycin and radiation therapy in squamous cell carcinoma of the upper aero-digestive tract: a phase III clinical trial. *Int. J. Radiat. Oncol. Biol. Phys.*, **11**, 1877.